# Frequency and Reasons for Fixation Hardware Removal After Orthognathic Surgery in Patients Treated in One Center

**DOI:** 10.3390/medicina61030403

**Published:** 2025-02-26

**Authors:** Rafał Nowak, Anna Olejnik, Szymon Przywitowski, Ewa Zawiślak, Paweł Golusiński

**Affiliations:** 1 Department of Otolaryngology and Maxillofacial Surgery, Institute of Medical Science, University of Zielona Góra, 65-046 Zielona Góra, Poland; 2 Face Surgery and Aesthetic Center, Pl. Powstańców Śląskich 1, 53-329 Wrocław, Poland

**Keywords:** orthognathic surgery, bone plates, surgical fixation devices, internal fixators

## Abstract

*Background and Objectives*: Despite the well-established position of orthognathic surgery as a field of surgical treatment of deformities within the facial skeleton, it has not been possible to develop unanimous recommendations on how to approach fixation hardware after the healing period. In the absence of clear guidelines from opinion leaders and scientific societies on how to approach osteosynthesis after surgery, the decision to leave or remove fixation hardware is made individually by treatment centers, mostly based on their own experience. It is also important whether or not surgical procedures are financed by public funds. This issue extends beyond orthognathic surgery, affecting all facial skeleton procedures involving osteosynthesis materials. The aim of this study is to analyze the frequency and reasons for fixation hardware removal after orthognathic surgery in patients treated in one center. *Materials and Methods*: This retrospective study examined the medical records from 2015 to 2020 of patients treated surgically for skeletal deformities at the Department and Clinic of Otolaryngology and Maxillofacial Surgery of Collegium Medicum (formerly the Otolaryngology Department of the Provincial Hospital in Zielona Góra). This study analyzed the age and sex of patients, the type of orthognathic procedure, and the type of skeletal deformity, as well as the reasons for fixation hardware removal in the groups of patients. *Results*: During this period, 124 orthognathic procedures were performed, including 56 one-jaw operations (BSSO or Le Fort I maxillary osteotomy), 2 one-jaw operations with genioplasty, 55 bimaxillary operations (BSSO + Le Fort I maxillary osteotomy), 6 bimaxillary surgery with genioplasty and 5 isolated genioplasty procedures. Fixation hardware was removed in 77 cases (62.10% of procedures), comprising 57 women and 20 men. Reasons for osteosynthesis removal were divided into three groups: complications such as the occurrence of inflammatory reaction/infection (*n* = 17), subjective discomfort (*n* = 23), and patient requests (*n* = 37). *Conclusions*: The findings underscore the need for scientific societies to establish unified guidelines on managing post-surgical fixation hardware to standardize care and enhance patient outcomes.

## 1. Introduction

Over the years, the methods and surgical techniques used to treat skeletal deformities have changed, but there is no doubt that three procedures have become firmly established in the canon of orthognathic surgery: sagittal osteotomy of the mandible (BSSO) using the Obwegeser technique with subsequent modifications, Le Fort I maxillary osteotomy, and chin plastic surgery or genioplasty [1].

Just as the methods themselves evolved, although the fundamentals remained the same, so too did the techniques and methods of immobilizing bone fragments after osteotomy. External immobilization in the form of intermaxillary wiring was originally used, but various types of osteosynthesis are now the standard.

Despite the introduction of osteosynthesis of bone fragments using plates and screws into orthognathic surgery in the 1970s, a consensus on the optimal approach to fixation hardware remains elusive.

In practice, there is no shortage of support for their removal [2,3,4,5,6] and for their retention after the primary operation [7,8,9].

In the event of clinical symptoms associated with fixation hardware, the treatment of choice is their removal. For fixation hardware that does not cause symptoms, the decision usually depends on the patient, the surgical team, or the experience of the clinical unit.

The Strasbourg Osteosynthesis Research Group (SORG) made the following recommendation in 1991: “A plate which is intended to assist the healing of bone becomes a non-functional implant once this role is completed. It may then be regarded as a foreign body. While there is no clear evidence to date that a plate causes actual harm, our knowledge remains incomplete. It is therefore not possible to state with certainty that an otherwise symptomless plate, left in situ, is harmless. The removal of a non-functioning plate is desirable provided that the procedure does not cause undue risk to the patient.” [10].

At the time of writing, a number of osteosynthesis kits are available for use in orthognathic surgery. Manufacturers are continually developing new solutions that are more effective than those already on the market. Modifications are being made to the shape and stiffness of the plates, as well as the diameter of the screws. Preformed plates are also available for a specific type of treatment. There are both screws that require pre-drilling and self-drilling screws, which only need to be screwed in without prior drilling. The gold standard is fixation hardware made of a titanium alloy, although in recent years there have been many attempts to use resorbable materials that would not require another procedure for their removal. The advent of three-dimensional planning technology and the printing of operating templates has led to the emergence of patient-specific osteosynthesis plates tailored to individual anatomical characteristics and the specifics of the planned surgical procedure.

Despite these advancements, there is still no universally accepted approach to the management of fixation hardware after bone healing. Some clinicians recommend routine removal due to potential complications such as infection, foreign body reactions, or long-term discomfort, while others emphasize the stability and biocompatibility of modern implants, arguing against unnecessary surgical interventions. In the absence of standardized guidelines, the decision regarding fixation hardware removal is often made on an individual basis, influenced by clinical experience, patient preferences, and institutional policies. Therefore, this study aims to analyze the frequency and underlying reasons for fixation hardware removal in a single-center cohort, providing insights that may contribute to the ongoing discussion about optimal post-surgical management strategies [11,12].

## 2. Materials and Methods

The present research study was conducted in accordance with the regulations of the Bioethics Committee University of Zielona Góra, which stipulate that scientific research involving the retrospective study of medical records does not require the opinion of the Bioethics Committee provided that the findings of such research do not influence the routine management of patients. Therefore, while we acknowledge the importance of ethical considerations in research, we followed the established guidelines of our institution for this particular type of study. This study complies with the World Medical Association Declaration of Helsinki on medical research protocols and ethics.

Our retrospective study examined the medical records of patients treated surgically for skeletal deformities at the Department and Clinic of Otolaryngology and Maxillofacial Surgery of Collegium Medicum (formerly the Otolaryngology Department of the Provincial Hospital in Zielona Góra) from 2015 to 2020.

From January 2015 to December 2020, 124 orthognathic procedures were performed, including 56 one-jaw operations (BSSO or Le Fort I maxillary osteotomy), 2 one-jaw operations with genioplasty, 55 bimaxillary operations (BSSO + Le Fort I maxillary osteotomy), 6 bimaxillary operations with genioplasty, and 5 isolated genioplasty procedures. The reason for treatment was a class III deformity in 80 cases, a class II deformity in 40 cases, an open bite in 3 cases, and a facial asymmetry in 1 case.

The necessity to remove fixation hardware after orthognathic surgery performed by the same team was adopted as the basic criterion for qualifying patients for the study group. Only cases where the primary indication for hardware removal was explicitly documented in the medical records were considered. Exclusion criteria included patients who sought hardware removal for reasons unrelated to orthognathic surgery, cases with incomplete medical records lacking documentation on the reason for removal, and patients with systemic conditions that could affect wound healing. Additionally, patients who underwent orthognathic surgery outside of our institution but later presented for hardware removal were excluded from the study. The fixation hardware removal procedures were performed by two experienced maxillofacial surgeons, and the technique of their application was similar.

After analyzing the medical records, it was found that between 2016 and 2022, the fixation hardware removal procedure was performed in 77 cases.

Medical data were obtained from the operation protocols as well as the patients’ case histories.

**Figure 1:**
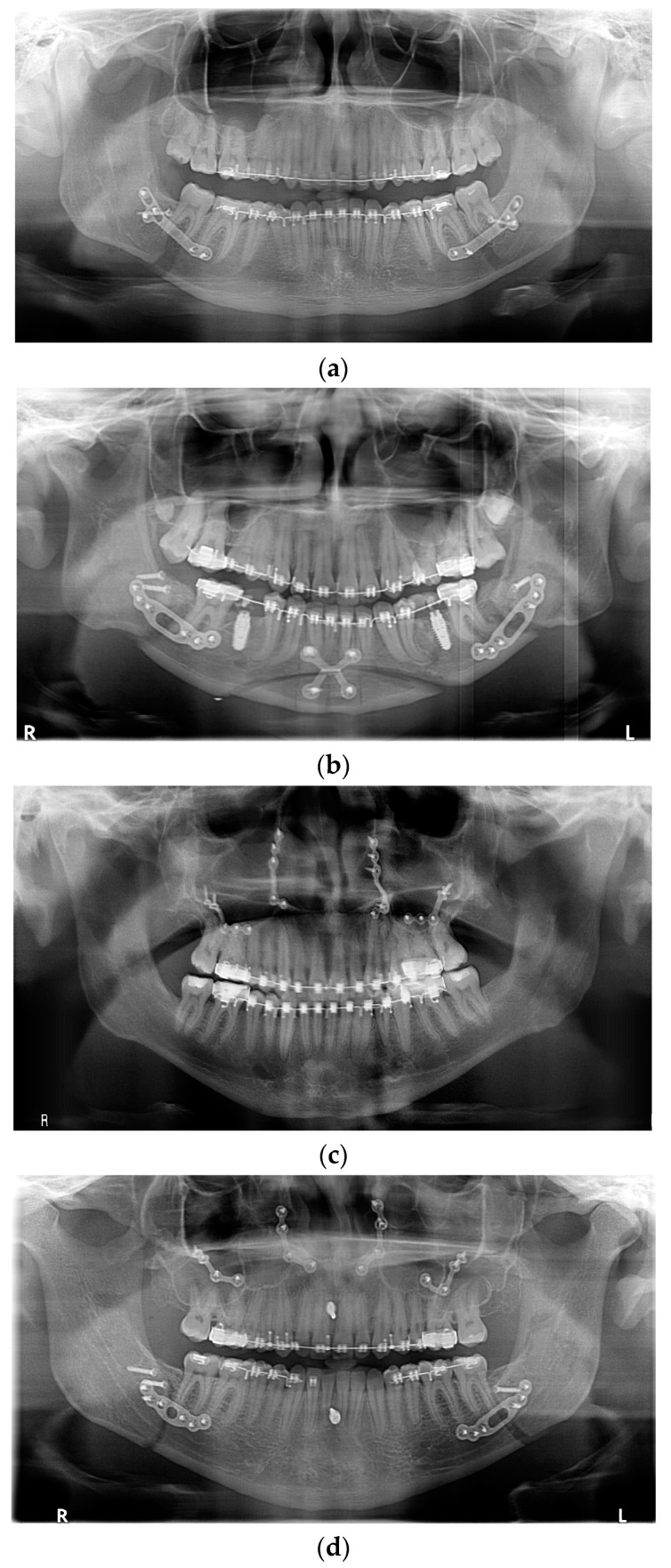

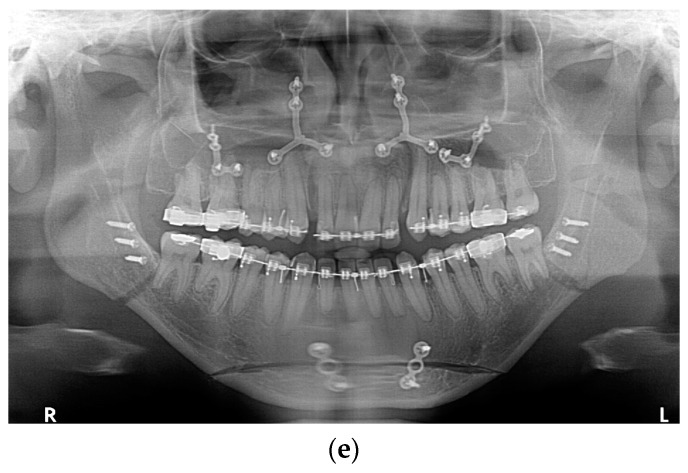
Panoramic X-ray with fixation hardware: (**a**) after BSSO; (**b**) after BSSO and genioplasty; (**c**) after Le Fort I osteotomy; (**d**) after bimax; (**e**) after bimax with maxillary segmentation and genioplasty.

All fixation hardware used in orthognathic procedures was made of titanium (Figure 1).

In the case of sagittal mandibular ramus osteotomy, the typical way to fix the osteotomy fragments was to use one bicortical screw as well as a single plate and monocortical screws on both the right and left side. In a few selected cases, only three bicortical screws were used without a plate, the use of a bicortical screw was abandoned and the fragments were provided fixation using only one plate, and monocortical screws or more than one bicortical screw and a plate were used. Each time it was dictated by individual anatomical and mid-operative conditions. All fixation hardware in the mandible was made with screws that required drilling and were made through an intraoral approach.

In Le Fort I maxillary osteotomy, the osteotomy fragments were typically fixed using 4 plates (most commonly in the shape of the letter “L”; for maxillary segmentation “Y” plates were used) and an appropriate number of monocortical screws. Screws that do not require drilling, i.e., self-drilling screws, were used. In selected situations dictated by anatomical conditions, customized solutions were used in the form of an additional plate, a non-standard shaped plate, or a reduced number of screws.

For genioplasty, dedicated plates with a bridge of appropriate length or two plates bent by the operator according to the planned bone movement were used together with monocortical screws.

In each case, regardless of the reason for fixation hardware removal, patients were informed of the technique, the risks associated with the procedure, and the recovery period and gave their formal written consent to the procedure.

The surgical procedure was performed under general anesthesia with nasal–tracheal intubation or oral–tracheal intubation and local anesthesia (mepivacainum 0.5% 10 mL) and perioperative antibiotic prophylaxis (2 g cefazoline i.v.). Mid-operatively, patients received 8 mg dexamethasone, and in justified cases, due to prolonged surgery or concomitant infectious complications, antibiotic therapy was continued until 5 days after surgery (amoxicillinum + acidum clavulanicum 1 g post-surgery or cefuroxime 0.5 g post-surgery). Patients were discharged from the hospital on the first day after surgery.

The data obtained from the records were entered into Numbers (Apple, Cupertino, CA, USA) to create a database and then subjected to statistical analysis using R version 4.4.2 (The R Foundation, Vienna, Austria).

The analysis of quantitative variables (i.e., expressed in numbers) was carried out by calculating descriptive statistics such as mean, standard deviation, median, quartiles, and minimum and maximum.

The analysis of qualitative (i.e., not expressed in numbers) variables was carried out by calculating the absolute and percentage rates of occurrence of all the values that these variables could take.

Comparisons of values for qualitative variables between the groups were made using the chi-squared test (with Yates correction for 2 × 2 tables) or the Fisher’s exact test when the chi-squared test assumptions regarding expected values were not met.

The comparison of quantitative variables in more than 2 groups was performed using the Kruskal–Wallis test and, when statistically significant differences between the groups were detected, the post hoc Dunn test was used.

The study used a significance level of 0.05, so all *p* values below 0.05 were interpreted as indicating significant relationships.

The age, sex of patients, type of orthognathic procedure, type of skeletal deformity, and reasons for fixation hardware removal in the groups of patients were analyzed.

## 3. Results

A total of 124 orthognathic procedures using osteosynthesis were performed and fixation hardware was removed in 77 patients, including 57 women and 20 men.

This represents 62.10% of 124 procedures performed.

The distribution of age and sex is presented in Table 1.

The reasons for osteosynthesis removal were divided into three groups.

**Figure 2:**
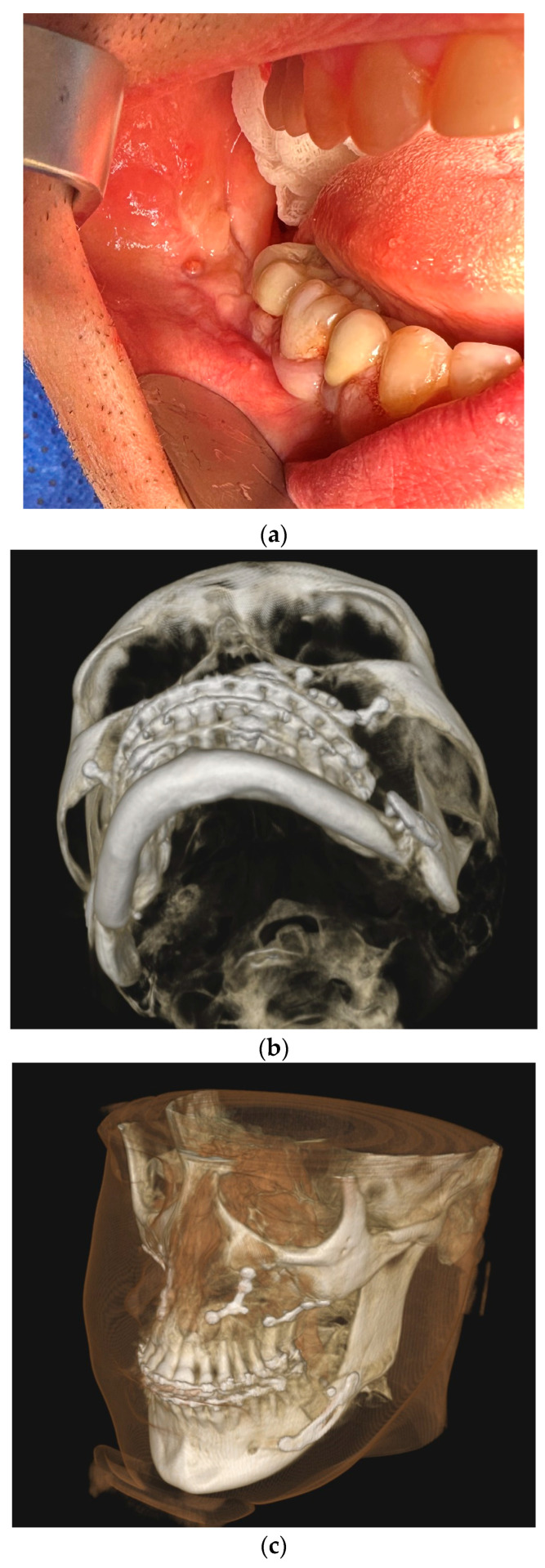
Image of inflammation/infection: (**a**) intraoral photo with fistula in the fixation area; (**b**) CBCT mandibular screws not fixed due to inflammation; (**c**) CBCT osteonecrosis in the bicortical screw area.

The first group included complications such as the occurrence of inflammation, infection, and fistula in the fixation area, swelling, or fixation exposure (Figure 2). This occurred in 17 cases, representing 22.08% of 77 fixation hardware removal procedures and 11.97% of 124 orthognathic procedures performed.

In instances of inflammatory complications or infections, the affected area in 11 cases was the maxilla and in 6 cases it was the mandible.

In 4 of 17 cases where complications were the reason for osteosynthesis removal, only the hardware that caused the problem was removed, and in the remaining 13 cases, all fixation hardware was removed at the same time.

**Figure 3:**
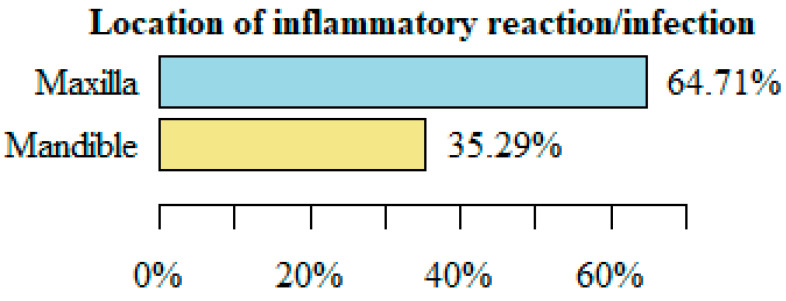
Location of inflammatory reaction/infection.

Figure 3 shows the location where the complication occurred—inflammation/infection associated with fixation hardware.

The second group of reasons included all subjective feelings of the patient related to the presence of fixation hardware. These feelings included the sensation of a foreign body, the sensation of cold when the ambient temperature changed, discomfort when touching the fixation site, and the sensation of mobility in the fixation area. These sensations were not accompanied by any clinical symptoms of inflammation or any other pathological processes. Due to subjective feelings of patients, fixation hardware was removed in 23 cases (29.87% of 77 fixation hardware removal procedures, which is 18.54% of 124 orthognathic procedures performed).

The third group included circumstances in which fixation hardware was removed at the explicit request of the patient without any objective and subjective clinical reasons. These were the most prevalent, accounting for 37 cases (48.05% and 29.83%, respectively).

Table 2 presents the reasons for osteosynthesis removal in the group of 77 patients who underwent such surgery classified according to sex, type of primary operation, and skeletal class. Using the *p* chi-squared or Fischer’s exact test, no statistically significant differences were found in either of these groups.

However, taking into account the number of originally performed orthognathic procedures (*n* = 124) classified according to the sex of patients (74 women and 50 men), statistically significant differences with regard to sex were found. Fixation hardware was statistically more often removed in women than in men for each of the listed reasons.

Table 3 shows the reasons for removing fixation hardware according to sex.

It was also found that fixation hardware was statistically more frequently removed in patients with skeletal class II: out of 40 patients, fixation hardware was removed in 31 cases (77.5%). In 80 patients with skeletal class III, fixation hardware was removed in 45 cases (56.25%).

In both skeletal classes, the percentage of inflammatory reasons for fixation hardware removal was similar: 12.5% in class II and 15% in class III.

For subjective reasons, fixation hardware was removed in 10 cases in class II (25%) and 13 cases in class III (16.25%), while there were clearly more cases of removal at the patient’s request in class II (40%) than in class III (25%).

**Figure 4:**
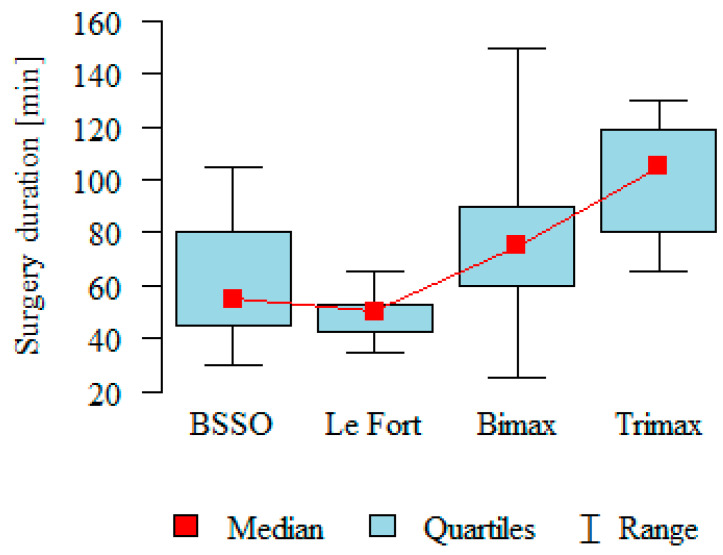
Length (minutes) of surgical fixation hardware removal.

The duration of the procedure for osteosynthesis removal was mainly dependent on the extent of the primary operation. As could be expected, procedures for removing osteosyntheses after one-jaw operations were shorter than after bimaxillary operations. It took less time to remove fixation hardware after Le Fort I jaw osteotomy than after sagittal/split mandibular ramus osteotomy (BSSO) (Table 4, Figure 4).

**Figure 5:**
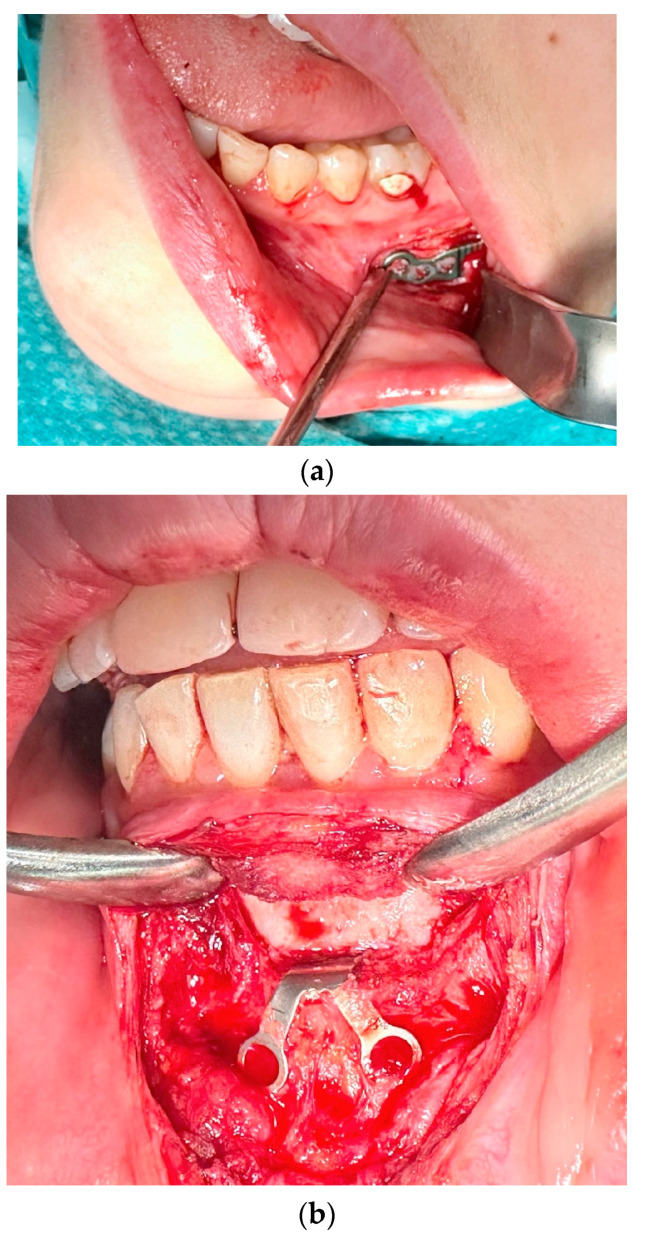

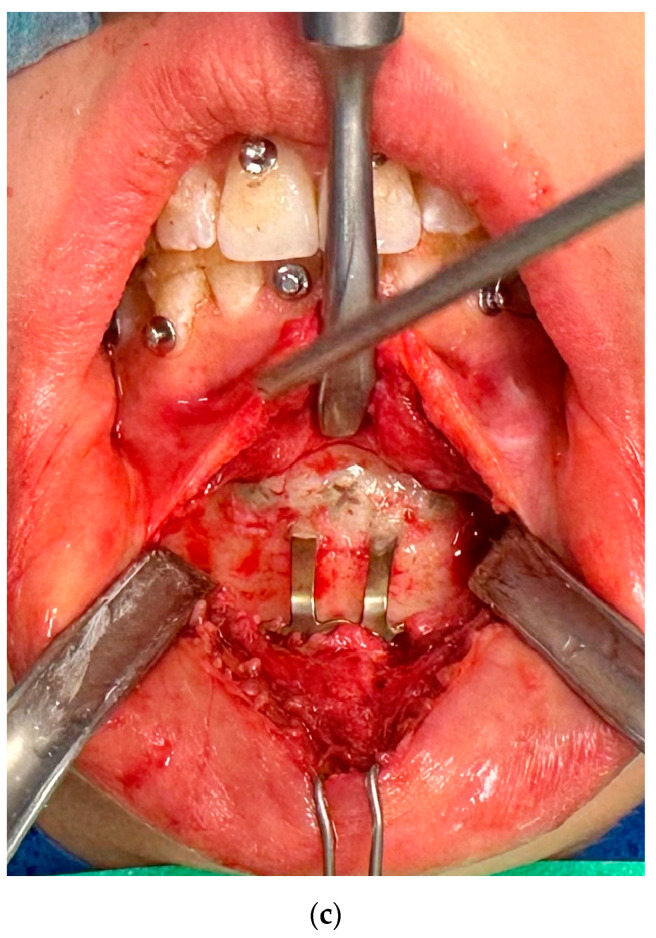
(**a**–**c**) Images of osteosynthesis covered with a layer of bone tissue.

The length of the surgical procedure was also influenced by the fact that in some cases, the fixation hardware was covered with a layer of bone tissue of different thickness and it was necessary to clean the screw slots, which required additional time and, as a result, prolonged the time of surgery (Figure 5).

This situation was recorded in 15 cases. In 11 cases, it was related to osteosynthesis in the mandible.

Table 5 shows the time from orthognathic surgery to fixation hardware removal.

This is consistent with the experience of other centers [9,12], as 44 procedures were performed between 6 and 12 months. At the earliest, we had to remove fixation hardware after 7 weeks because of persistent acute inflammation, and at the latest, 59 months after the primary operation.

In four cases, septoplasty was performed simultaneously with osteosynthesis removal. In two cases, septoplasty was performed, and in one case, mandibular body reconstruction was performed.

## 4. Discussion

Before Hugo Obwegeser pioneered modern orthognathic surgery in the 1950s, numerous surgical techniques were employed to cut the deformed bone and reposition the bone fragments. The common feature of these procedures was the use of external immobilization either in the form of rigid maxillary wiring or with the use of various orthopedic devices. The concept of direct bone fragment immobilization was first developed in the field of maxillofacial traumatology, which in turn originated in orthopedics.

The basic principle of treating fractures is to position them correctly and to use internal or external immobilization to hold them in the position obtained until bone adhesions are formed.

Various types of external immobilization have been used for long bone fractures since antiquity, and the concept of open treatment using the technique of direct fixation of bone fragments only emerged in the 19th century. Similarly, until the 19th century, external immobilization with bandages, wire ligatures, or various types of chin straps was the usual method of treating craniofacial injuries.

The first successful fixation of long bone fragments using silver wire was performed by the New York surgeon Rodgers in 1827, while the first known surgical treatment of a mandible fracture was the placement of circumferential wiring by the French military surgeon Baudens in 1840 [1].

The author of the term “osteosynthesis” was Albin Lambotte, who also introduced a set of instruments and aluminum plates to immobilize mandibular fragments. Sir Arbuthnot William Lane is also considered to have pioneered the use of direct fixation in the treatment of fractures [13].

According to some sources, Hansmann first described the use of a metal plate to treat jaw fractures in 1886 [13], and other sources report that the first plate fixation was performed by the German surgeon Soerensen in 1917, using a wedding ring [14].

A real revolution in the field of providing fixation to bone fragments in the skull occurred in the 1970s with the introduction of treatment using miniaturized plates [3,15] and the knowledge of the most advantageous sites for their placement in the bone [2]. It was not long before these techniques were used in orthognathic surgery. For the first time, bicortical screws were used by Spiessl in 1974 [16] to join mandibular bones after osteotomy.

Although these techniques made it possible to eliminate the need for intermaxillary wiring after surgery, thereby facilitating greater control over the airways, they were initially met with criticism from the surgical community dealing with skeletal deformities, with Hugo Obwegeser himself being a prominent opponent.

However, clinical observations of fixation using miniplates and screws provided a more stable adhesion of bone fragments than previously used techniques, which meant that nothing stood in the way of further development of osteosynthesis.

The first osteosynthesis kits were made from various alloys containing steel, chromium, and cobalt. In the 1980s, titanium and its alloys were introduced as the main material for the production of plates and screws.

Due to its properties such as biocompatibility, osteoinduction, and osteoconduction; elasticity comparable to that of compact bone; high mechanical strength; no electrical or thermal conductivity; no magnetic field influence; low weight; and low corrosivity, titanium seems to be an ideal material for bone fixation [17,18].

The compatibility of titanium with human tissues has been demonstrated in both in vivo and in vitro studies. However, concerns have been raised regarding the long-term effects of local and general titanium retention in the human body.

The existing literature reveals that there are reports indicating that pure titanium does not exert any influence on carcinogenesis, inflammation, or allergic reactions [7]. Nevertheless, there are also reports indicating a correlation between chronic fibrous tissue inflammation and the presence of titanium. The presence of titanium molecules in the lymph nodes was also observed [19,20].

In the absence of clear guidelines from opinion leaders and scientific societies on how to approach postoperative osteosynthesis, the decision to leave or remove fixation hardware is made individually by treatment centers.

In the case of fixation hardware that is causing inflammation or other objective discomfort, it is clear that it must be removed. However, in the case of fixation hardware that causes no discomfort, the decision is based on experience.

Some centers advocate for the routine removal of plates [4,21]. This is corroborated by the potential for complications to emerge due to the presence of specific bacterial flora in the oral cavity and the influence of masticatory forces. The presence of teeth and potential odontogenic foci also increases the risk of inflammatory complications in the event of infection.

Other authors recommend observation and action in the event of clinical symptoms [7,22,23,24].

In the course of our study, we removed fixation hardware in 77 cases out of 124 orthognathic procedures performed, which represents 62.10% of the total number of cases. This is a high percentage compared to the data available in the literature [9,11,12,22,23,24,25,26,27,28,29,30,31], where the percentage varies from 2% to 55%.

This may be due in part to the fact that patients are routinely informed of the possibility of fixation hardware removal prior to orthognathic surgery, and there is still a lack of consensus in the medical community on how to approach osteosynthesis after healing. Patients prepared for orthognathic surgery in our team are informed about the risks associated with the removal of fixation hardware and the fact that the asymptomatic course in the initial period after orthognathic surgery does not guarantee that fixation hardware will not cause problems in the long term. We also inform patients that the optimal time for surgery is 6–12 months after the primary operation and that the planned osteosynthesis removal procedure is performed under general anesthesia. However, it is important to bear in mind the growing awareness of patients in the age of information sharing enabled by the Internet.

In our study, almost 30% of fixation hardware was removed at the explicit request of the patient, without any clinical symptoms or subjective feelings of the patient, and the main argument for surgery was to avoid future complications. Sukegawa et al. [26] reported that fixation hardware was removed at the patient’s request after orthognathic procedures in 19.6% of all cases, while Park et al. [27] also reported the patient’s request as the main reason for osteosynthesis removal.

The second most common reason for fixation hardware removal was the presence of discomfort in the absence of evident inflammatory symptoms or infection. In this case, it was not considered a complication but a subjective feeling of the patient. In our group, this cause was the reason for 18.54% of all interventions. For this reason, Parasher et al. [28], in their study on a group of 352 patients, removed fixation hardware in 24 cases, or 6.81% of all patients.

The third reason, inflammation, infection, or fixation exposure in our material, occurred in 17 cases, or 11.97%. It is therefore widely accepted that osteosynthesis removal is a necessary step in the treatment of this complication. According to reports in the literature, the percentage of cases in which it is necessary to remove fixation hardware for inflammatory and/or infectious reasons ranges from 2.8% [25] to 22.2% [9].

There is a tendency for fewer inflammatory complications when bicortical screws are used to provide fixation for osteotomy fragments in the mandible than when plates are used [9,25,29,30,31,32].

There have also been reports of potential bacterial colonization of osteosynthesis materials by blood, which, despite the lack of clinical symptoms in the initial postoperative period, may be the reason for the need for intervention in the future [32].

In a meta-analysis of fixation hardware removal in orthognathic surgery, Gomez-Barrachina et al. [33] demonstrated that indications for re-intervention were present in 13.4% of all cases. Furthermore, they identified female sex as a risk factor, in addition to the two aforementioned factors.

In our material, complications related to fixation hardware in the maxilla were more frequent, which differs from the above reports, while in our patients, complications occurring in the fixation area in the mandible were much more clinically serious than those occurring in the maxilla.

Statistically, we removed fixation hardware more frequently in women (77.03% vs. 40%, Table 3) both for inflammatory/infectious reasons and at the patient’s request. This is consistent with previous research suggesting that female patients may be more likely to request hardware removal due to subjective factors such as sensitivity, pain, palpability, or psychological discomfort [34].

Similarly, Falter et al. reported a significantly higher rate of hardware removal in female patients (31.7%) compared to male patients (20.3%) (*p* = 0.0091), with differences in the indications for removal. While the rates of removal due to infection were comparable between sexes, female patients were significantly more likely to undergo removal due to clinical irritation (15.2% vs. 5.3%), further supporting the hypothesis that subjective factors may contribute to their decision [35].

Furthermore, our findings align with Gomez-Barrachina’s observations that the female sex shows a greater predisposition to inflammatory complications associated with osteosynthesis [33]. However, the influence of factors such as differences in pain perception, heightened body awareness, or sociocultural expectations regarding medical interventions requires further investigation. Among the complications after the procedure of fixation hardware removal, the authors mentioned intraoperative and postoperative bleeding and sensory disorders, which occurred in 23 cases (9.87%).

In three of our patients, healing was complicated by the formation of hematoma and associated significant swelling, which caused discomfort to the patients in the postoperative period and required prolongation of antibiotic therapy as well as the use of non-steroidal anti-inflammatory drugs.

Many authors agree that fixation hardware removal after maxillofacial surgery should take place within 1 year of the primary operation [36,37]. In the study group, the most frequent removal of fixation hardware occurred between 6 and 12 months after orthognathic surgery, although some patients decided to undergo elective surgery after more than 12 months. In six cases, the need to remove osteosyntheses before 6 months after surgery was due to the clinical situation—developing inflammation.

Resorbable plates have been introduced as an alternative to titanium fixation hardware to eliminate the need for a second operation for plate removal [33,38,39]. While the use of resorbable materials appears to offer certain advantages, such as reducing the long-term presence of foreign bodies and avoiding additional surgical interventions, concerns remain regarding their stability, degradation process, and potential complications.

A multi-center randomized controlled trial by van Bakelen et al. found that the risk of plate removal in patients treated with biodegradable fixation systems was 2.2 times higher than in those treated with titanium plates, with abscess formation being the most common reason for removal [38].

Similarly, a review comparing resorbable and titanium plates reported a higher removal rate for resorbable plates (3.63%) compared to titanium (1.53%), with the main complications being infection, plate exposure, and sinus tract formation [39].

Given these findings, while resorbable plates offer theoretical advantages, their higher removal rate and increased risk of complications, particularly in mandibular osteotomies, raise concerns about their routine use in orthognathic surgery.

A major limitation of this study is its retrospective design, which inherently introduces potential biases, particularly in patient-reported outcomes. Since data were obtained from medical records rather than prospective patient follow-up, factors influencing the decision to remove fixation hardware may not have been fully captured. Additionally, this study was conducted at a single center, which limits the generalizability of the findings to other populations and institutions with different surgical protocols or healthcare policies.

Another limitation is the lack of a comparative follow-up for patients who retained their fixation hardware. Without long-term outcome data for patients who did not undergo hardware removal, it remains unclear whether their long-term satisfaction or complication rates differ significantly from those who opted for removal. Future prospective studies, ideally randomized trials, are needed to assess patient satisfaction and long-term outcomes in both groups.

Furthermore, while we analyzed sex differences in hardware removal rates, we did not account for other potential confounding variables, such as patient comorbidities, socioeconomic factors, or surgical variations, which could have influenced the decision for hardware removal. Addressing these factors in future multi-center studies could provide a more comprehensive understanding of the determinants influencing fixation hardware management.

## 5. Conclusions

The results of this study indicate that the percentage of fixation hardware removal in our cohort was higher than what has been reported in the literature, with patient preference being the most common reason for removal. Additionally, women were more likely to undergo hardware removal than men, which may be associated with increased sensitivity to discomfort or greater awareness of potential long-term complications. These findings highlight the variability in current clinical practice and underscore the need for clearer guidelines on post-surgical fixation hardware management.

From a clinical standpoint, the decision to remove fixation hardware should be individualized, taking into account the presence of symptoms, risk factors for complications, and patient preferences. Given the higher removal rates observed in women, particular attention should be paid to understanding the factors influencing their decision making.

Further research is needed to evaluate long-term outcomes in patients who retain their fixation hardware compared to those who undergo removal. Prospective, multi-center studies, ideally including randomized controlled trials, could provide stronger evidence to guide clinical decision making. Additionally, further advancements in biodegradable fixation materials should be explored to determine whether they can serve as a viable alternative to titanium implants and potentially reduce the need for secondary procedures.

## Figures and Tables

**Table 1:** Sex and age.

Parameter		Total (N = 77)
sex	female	57 (74.03%)
	male	20 (25.97%)
age [years]	mean (SD)	29.62 (5.7)
	median (quartiles)	29 (25–34)
	range	20–45
	*n*	77

**Table 2:** Sex, primary surgery, skeletal class and the reason for osteosynthesis removal.

Parameter		Reason for Osteosynthesis Removal			*p*
		Inflammatory Reaction/ Infection (N = 17)	Subjective Patient Feelings (N = 23)	Patient’s Request (N = 37)	
sex	female	11 (64.71%)	16 (69.57%)	30 (81.08%)	*p* = 0.387
	male	6 (35.29%)	7 (30.43%)	7 (18.92%)	
orthognathic procedure	one-jaw surgery	3 (17.65%)	5 (21.74%)	16 (43.24%)	*p* = 0.112
	one-jaw surgery with genioplasty	1 (5.88%)	0 (0.00%)	0 (0.00%)	
	double-jaw surgery	11 (64.71%)	15 (65.22%)	20 (54.05%)	
	double-jaw surgery with genioplasty	2 (11.76%)	3 (13.04%)	1 (2.70%)	
skeletal class	class II	5 (29.41%)	10 (43.48%)	16 (43.24%)	*p* = 0.555
	class III	12 (70.59%)	13 (56.52%)	20 (54.05%)	
	asymmetry	0 (0.00%)	0 (0.00%)	1 (2.70%)	

*p* chi-squared or Fisher’s exact test.

**Table 3:** Reason for fixation hardware removal (*n* = 77) according to sex in a group of 124 patients who underwent orthognathic surgery.

Parameter		Sex		*p*
		Female (N = 74)	Male (N = 50)	
osteosynthesis removal	yes	57 (77.03%)	20 (40.00%)	*p* < 0.001 *
	no	17 (22.97%)	30 (60.00%)	
reason for osteosynthesis removal	inflammatory reaction/infection	11 (14.86%)	6 (12.00%)	*p* < 0.001 *
	subjective patient feelings	16 (21.62%)	7 (14.00%)	
	patient’s request	30 (40.54%)	7 (14.00%)	
	no removal	17 (22.97%)	30 (60.00%)	

*p* chi-squared or Fisher’s exact test, * statistically significant (*p* < 0.05).

**Table 4:** Length (minutes) of surgical fixation hardware removal.

Procedure	N	Surgery Duration [min]							*p*
		Mean	SD	Median	Min	Max	Q1	Q3	
BSSO—A	17	61.18	22.26	55	30	105	45.0	80.00	*p* < 0.001 * D, C > A, B
Le Fort—B	7	48.57	9.88	50	35	65	42.5	52.50	
bimax—C	46	82.50	29.70	75	25	150	60.0	90.00	
trimax—D	6	100	26.08	105	65	130	80.0	118.75	

*p*—Kruskal–Wallis test + post hoc analysis (Dunn test), SD—standard deviation, Q1—lower quartile, Q3—upper quartile; * statistically significant (*p* < 0.05).

**Table 5:** Time from primary operation to fixation hardware removal and the reason for osteosynthesis removal.

Time		Total (100%)	Inflammatory Reaction/ Infection	Subjective Patient Feelings	Patient’s Request
months	<6	6 (7.79%)	6	0	0
	6–12	44 (57.14%)	9	13	22
	>12	27 (35.06%)	2	10	15
	*n*	77	17	23	37

## Data Availability

The original contributions presented in this study are included in the article. Further inquiries can be directed to the corresponding author.
